# Formative research to inform development of a new diagnostic for soil-transmitted helminths: Going beyond the laboratory to ensure access to a needed product

**DOI:** 10.1371/journal.pntd.0007372

**Published:** 2019-05-31

**Authors:** Helen L. Storey, Neha Agarwal, Jason Cantera, Allison Golden, Kerry Gallo, Tara Herrick, Vicente Belizario, Jimmy Kihara, Charles Mwandawiro, Bill Cadwallader, Tala de los Santos

**Affiliations:** 1 PATH, Seattle, Washington, United States of America; 2 University of the Philippines, Manila, Philippines; 3 Kenya Medical Research Institute, Nairobi, Kenya; Brock University, CANADA

## Abstract

Soil-transmitted helminths (STHs) affect more than 1.5 billion people. The global strategy to control STH infections requires periodic mass drug administration (MDA) based on prevalence among populations at risk determined by diagnostic testing. Widely used copromicroscopy methods to detect infection, however, have low sensitivity as the prevalence and intensity of STH infections decline with repeated MDA. More sensitive diagnostic tools are needed to inform program decision-making. Using an integrated product development process, PATH conducted qualitative and quantitative formative research to inform the design and development of a more sensitive test for STH infections. The research, grounded in a conceptual framework for ensuring access to health products, involved stakeholder analysis, key opinion leader interviews, observational site visits of ongoing STH surveillance programs, and market research including market sizing, costing and willingness-to-pay analyses. Stakeholder analysis identified key groups and proposed strategic engagement of stakeholders during product development. Interviews highlighted features, motivations and concerns that are important for guiding design and implementation of new STH diagnostics. Process mapping outlined current STH surveillance workflows in Kenya and the Philippines. Market sizing in 2016 was estimated around half a million tests for lower STH burden countries, and 1–2 million tests for higher STH burden countries. The cost of commodities per patient for a molecular STH diagnostic may be around $10, 3–4 times higher than copromicroscopy methods, though savings may be possible in time and staffing requirements. The market is highly price sensitive as even at $5 per test, only 27% of respondents thought the test would be used by surveillance programs. A largely subsidized STH control strategy and a semi-functional Kato-Katz test may have created few incentives for manufacturers to innovate in STH diagnostics. Diverse partnerships, as well as balancing needs and expectations for new STH diagnostics are necessary to ensure access to needed products.

## Author summary

Soil-transmitted helminths affect more than 1.5 billion people, mostly in very poor regions without proper sanitation. To control infections, countries periodically give deworming drugs to populations at risk, such as school-age children, based on the results of diagnostic testing to determine the prevalence of infection. The current method to detect infection works when infections are prevalent, but is less useful after repeated deworming, when disease prevalence is lower. We conducted formative research—including user and market research—to inform development of a new diagnostic tool with improved sensitivity. Findings from this research highlight pain points with current methods and processes, which are also opportunities for innovation. Access to new STH diagnostics may improve program decision-making and eventually contribute to elimination of these infections.

## Introduction

Soil-transmitted helminths (STHs) remain a massive global health problem. Each year, more than 1.5 billion people suffer from infection with these parasitic intestinal worms [1]. STH infections have significant harmful effects on the health and well-being of individuals, especially children, in whom infection may lead to malnutrition, anemia, abdominal pains, stunted growth, and poor cognitive development [2–4]. The focus of the global STH control strategy is reducing morbidity in the most at-risk populations—such as preschool-age children, school-age children, and women of reproductive age—through mass drug administration (MDA) of donated deworming drugs, such as mebendazole and albendazole [5]. Program decisions for use of MDA currently rely on detecting the presence and load of helminth eggs in stool samples from at-risk groups. The recommended tests for measuring STH infections during surveillance activities involves microscopic examination of stools for STH eggs by Kato-Katz (KK) or mini-FLOTAC methods [6]. Although both copromicroscopy methods are adequate to support program decisions when the prevalence and intensity of infections are moderate to high, they lack sensitivity with light-intensity infections, especially after MDA has reduced infections to low levels [7–10].

**Fig 1:**
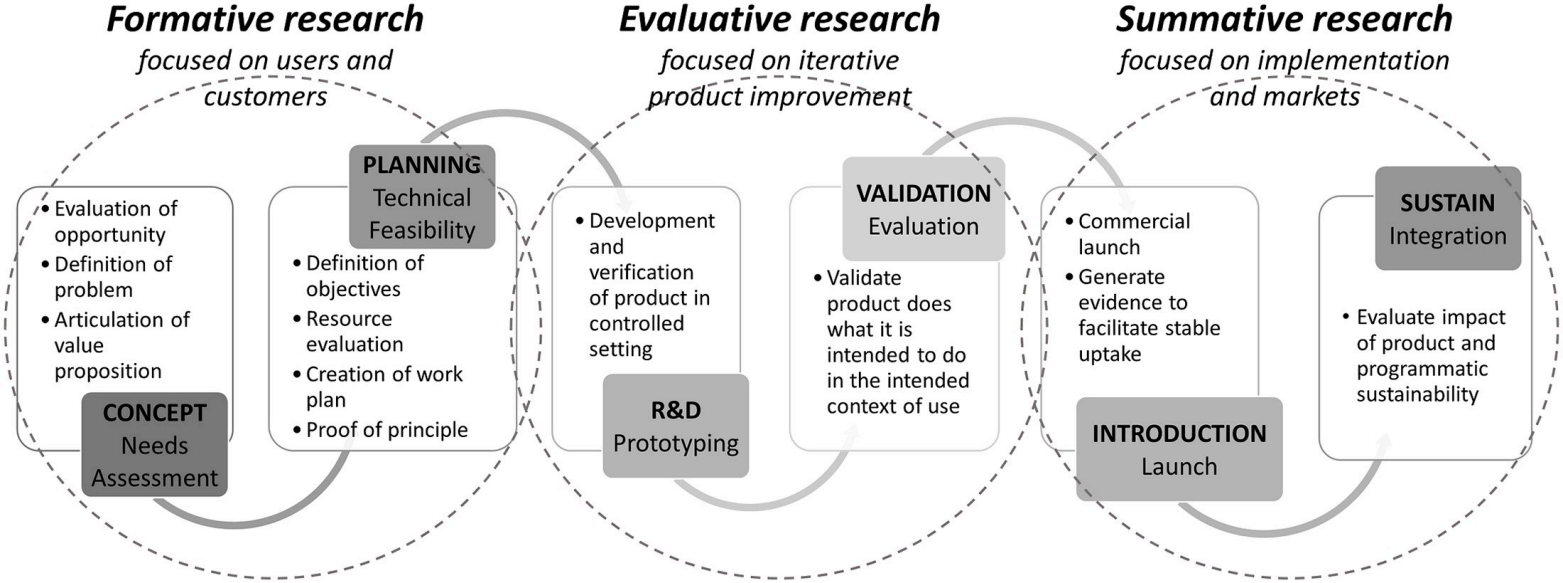
An integrated product development process requires research beyond the laboratory to ensure access to needed health products.

PATH supported the efforts of the 2012 London Declaration on Neglected Tropical Diseases (NTD) by conducting a diagnostic gap analysis for 9 NTDs: STH, Schistosomiasis, Onchocerciasis, Lymphatic Filariasis, Trachoma, Chagas disease, Leprosy, Visceral Leishmaniasis, and Human African Trypanosomiasis [11]. Based on findings of a lack of sufficient diagnostic tools to address the current control strategy for STH infections, further research and development on a new, more sensitive diagnostic tool was initiated. Building on the identified use cases and findings from the previous STH diagnostic gap analysis, the dominant need was a diagnostic tool to support decision-making for reducing or discontinuing MDA when the intensity of STH infection is low [12]. Concurrently, health impact modelling was performed and suggested that a more sensitive diagnostic could potentially add the greatest value in this setting [13]. By accurately detecting low-intensity infections, a new test may not only help to improve decision-making related to use of MDA but also contribute toward the eventual elimination of STH infections. PATH’s integrated product development process includes an early consideration for user and customer needs, as well as implementation constraints and market dynamics [14]. (Fig 1) Planning for sustainable access throughout the product lifecycle is imperative to achieving public health impact [15]. To overcome the shortcomings of current diagnostic methods for guiding MDA use, PATH conducted formative research to inform the design and development of a new, more sensitive test. This work included both user and market research and evaluated factors such as stakeholders and policies, impact and affordability, and user needs and acceptability.

**Fig 2:**
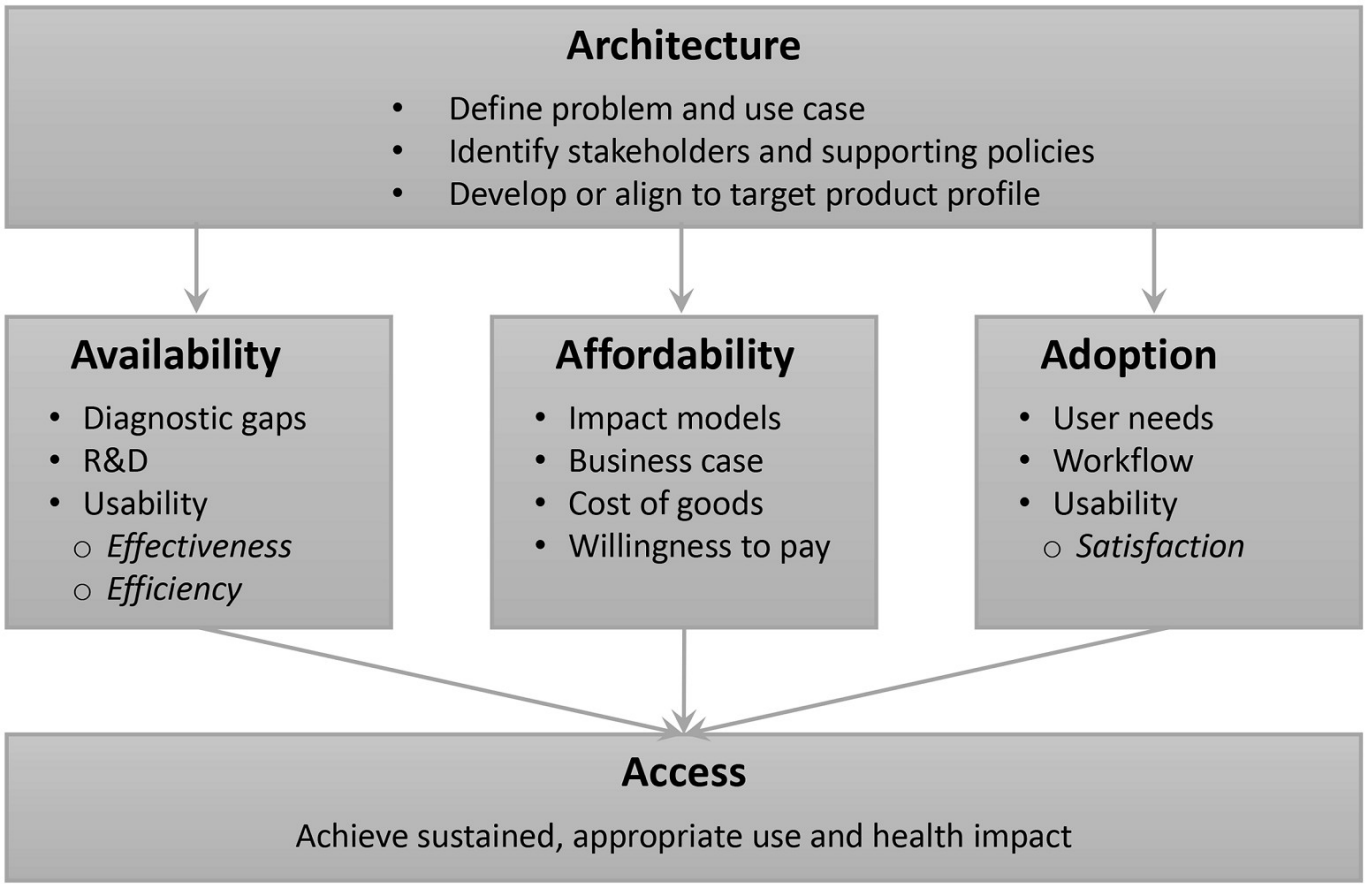
Important factors for ensuring access to new innovations: A focus on new diagnostics for measuring soil-transmitted helminth infections (adapted from Reich and Frost, 2008).

The Access Framework developed by Reich and Frost provided a conceptual framework for this formative research, which identifies four important factors in moving promising innovations across the value chain from development to large-scale impact: architecture, availability, affordability, and adoption [16]. (Fig 2) Key principles of this framework include [17]:

- Access necessitates “the right product at the right place with the right protocol at the right time”, but that alone is not sufficient to improve health, especially in poor countries.
- Planning for access requires understanding the facilitators, barriers, and key actors involved in supporting architecture, availability, affordability, and adoption.
- Because conventional market approaches often fail to ensure access to products in low-resource settings, creating cross-sector partnerships is essential for success.
- Identifying and addressing factors that influence adoption from the user’s perspective is critical as health seeking behaviors are often involved.
- “Expert consensus” on the need and use of a health product is an important facilitator of access, requiring an active process to produce acceptance.

The findings from this formative research informed PATH’s development of a new molecular diagnostic test for STH infections. The new test, named Dx4STH for easier reference, is a molecular diagnostic kit product that is rapid and sensitive, using a type of isothermal nucleic acid amplification called recombinase polymerase amplification (RPA) to detect four key STH species. Technical aspects of the product development will be discussed elsewhere. The focus here is on the methods and results of the formative research, and the research findings’ influence on PATH’s product development activities. This work may also serve as a foundation for any future research on STH diagnostics to ensure access to needed products.

## Methods

Formative research using qualitative and quantitative methods was conducted with the objective to investigate factors that could facilitate or hinder end-users’ access to a new, more sensitive test for STH infection. This formative research was conducted before and during product development from roughly 2014 to 2017. This study was reviewed by the PATH Research Ethics Committee and determined to be not human subjects research.

Early on, a stakeholder analysis was conducted focused on the development and implementation of STH diagnostics. Stakeholder analysis is a “process of systematically gathering and analyzing qualitative information to determine whose interests should be taken into account when developing and/or implementing a policy or program”[18], or product development project in this case. It is also a means to capture the unique attributes of a stakeholder audience, to better communicate and share information about the product or project that is tailored to their unique interests or concerns. Stakeholders were identified through a review of peer-reviewed literature, grey literature, World Health Organization (WHO) policies, organization websites, and participant lists from relevant recent meetings. Assessments by the research team were made of their knowledge, interest, position, and ability to effect change, using a three-point scale based on perceived influence and interest. Stakeholders were also grouped into categories (e.g., manufacturer, donor) based on their roles in the development and use of STH diagnostics. The search continued until no new categories were identified. Because stakeholders could fall into more than one category, the analysis was performed at the category level and resulted in a matrix to guide a participatory, consensus-building product development process.

Next, semi-structured interviews were conducted with key opinion leaders to better understand STH surveillance needs and activities in relation to surveillance for other neglected tropical diseases (NTDs). These interviews focused on stakeholders involved in programmatic aspects of NTD surveillance. A purposive, snowball sampling strategy was used to obtain a heterogeneous range of responses until saturation was reached [19]. Of the 14 individuals contacted, 10 interviews were conducted. Interview topics included the role of programmatic personnel in surveillance, current NTD surveillance activities, and STH surveillance and tools, including issues related to specimen collection, populations surveyed, instrument platforms used, and time to result. The individuals interviewed had experience with NTD surveillance programs in Africa, Asia, and the Americas. Interviews were recorded, transcribed, and then analysed using thematic content analysis. A codebook was created based on *a priori* themes and revised during analysis based on emerging themes. Data were analysed by one researcher and reviewed by the research team to discuss findings.

Ethnographic research in the form of observational site visits were performed to create process maps for government-led STH surveillance in Kenya and the Philippines [19]. PATH researchers identified and shadowed Ministry of Health-led teams conducting ongoing STH surveillance. Observations were captured using the AEIOU heuristic, which is a method to categorize data based on activities, environments, interactions, objects, and users [20]. Surveillance process maps were drafted, reviewed by field managers, and revised based on feedback. Key existing policies were also identified that support the use of STH diagnostics in the surveillance processes observed.

Market research was conducted to quantify potential markets, forecast health and economic impacts of using more sensitive STH diagnostics, estimate costs of goods, and assess willingness to pay. Findings from the health and economic impact modelling can be found elsewhere [13]. To quantify the potential market, country-specific data were used to estimate the total number of tests needed worldwide. Data from United Nations, Department of Economic and Social Affairs was used to determine the total number of children ages 5–14 years per country in 2013 and 2016 [21]. Data from the WHO STH data repository was used to determine the number of SAC requiring PC by country [22]. Calculations were originally determined with data from 2012, and then updated with 2016 data. Based on WHO guidelines, it was assumed that countries would conduct testing in five to ten schools per district, sampling 50 students per school, across the entire population of SAC because STH infections are not focally distributed [23]. The number of districts per country was calculated by dividing the total number of SAC by 200,000 as recommended in the guidelines for sentinel site monitoring. To create a standard method for approximating STH prevalence, the proportion of school-age children requiring PC for STH was determined by comparing the number of SAC requiring PC to the total number of children ages 5–14 years in the country. (S1 File)

A cost analysis was performed for 2 copromicroscopic methods and 4 molecular methods for detecting STH. Data for the Kato-Katz and FLOTAC method was derived from a 2010 publication and adjusted for 2016 costs [24]. For both the Kato-Katz and FLOTAC methods, the costs of instruments were considered primarily microscopes and assumed to be sunk costs as they already exist in most facilities and have a long life-span, thus they are not included in the analysis. The molecular methods included 2 product concepts in development at PATH, one using Dx4STH with the common DNA extraction kits used with qPCR, and a second method using Dx4STH with a simpler, prototype DNA extraction method that involves a modified alkaline lysis-magnetic bead protocol (MAL-MB). The prototype MAL-MB protocol was developed as a simpler method for DNA extraction from stool requiring less equipment and consumables. Multiparallel qPCR and multiplex qPCR were also included as they were conducted during product development as reference assays. For the molecular methods, all instrument costs including ancillary laboratory equipment such as centrifuges, magnetic racks, and heat baths were incorporated making a conservative assumption that these would be new instruments added to existing facilities where surveillance would take place. Additionally, optimal workflows and staffing for the molecular methods were determined based on protocols used in the PATH laboratory. (S2 File). Estimates were derived for roughly 100 samples per day, based on WHO guidelines. Summary costs were calculated by adding up component costs for instruments and consumables, then dividing by throughput. Staff time and infrastructure costs were not included for any methods, as comparable estimates across the 6 methods were not available. To account for the accuracy of the method, the total cost per accurate diagnosis was determined by dividing the total cost by the accuracy of the method. All details for the costing analysis are provided as supporting information. (S2 File)

A willingness-to-pay analysis was performed using both a perceived-value pricing (PVP) approach and a Gabor-Granger approach [25]. The PVP approach assesses pricing expectations and perceived usefulness of a new STH diagnostic using a scale between 0–100, 0 indicating “not at all useful” and 100 indicating “extremely useful”. Plotting perceived usefulness against the perceived price estimates the perceived value for money without reference to specific price points. Respondents were provided product primers to inform their assessments. To assess the price sensitivity of the market for a new STH diagnostic, the Gabor-Granger approach was used as a price experiment method [25]. The Gabor-Granger method uses a 5-point scale to assess the highest price a respondent would be willing to pay based on a range of fixed prices shown to the respondent. The two methods were used in combination to more accurately capture potential differences between customers and users. Data for these methods was prospectively collected using semi-structured interviews with 29 respondents across a range of stakeholder groups, and 15 countries in Africa, Asia, and the Americas. This analysis was not designed for statistical significance and the target sample size was 20–35 respondents to achieve a heterogeneous distribution of experience and geographical spread. Participation in any or all questions was voluntary, and no personal identifiable information was shared with the research team. These interviews built on, but were separate from the initial stakeholder interviews conducted, and were focused on assessing the commercial viability of the Dx4STH method.

## Results

### Stakeholder analysis

**Fig 3:**
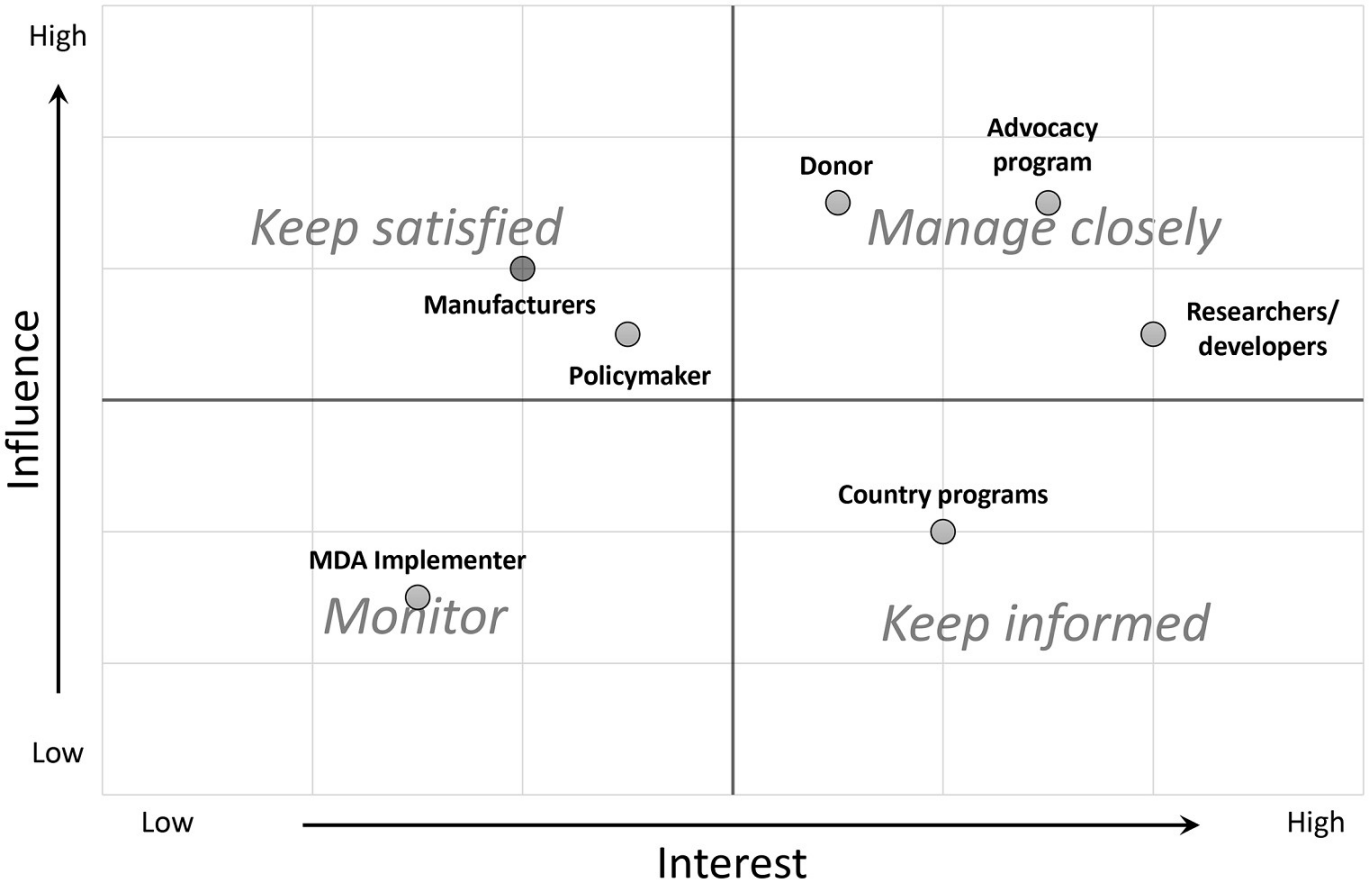
Using a stakeholder analysis to tailor communication strategies and guide a participatory, consensus-building product development process. Assessment of influence and interest of seven identified categories of stakeholders involved in the development and introduction of STH diagnostics, from the perspective of a product development partner.

A total of 70 stakeholder organizations were identified and grouped into seven categories: Donor, Manufacturer, MDA Implementer, Country Program, Researcher/Developer, Policymaker, and Advocacy Program. Because the stakeholder assessment is from the perspective of a product development partner, this category is not included. Advocacy Program was classified as both highly influential and interested because of its role as a champion for change and scientific advancement. Researcher/developer was classified as having medium influence and high interest because years of investigation by dedicated scientists have led to many advances in STH diagnostics despite limited funding. Donors were classified as highly influential with medium interest because donors fund most research and development for STH diagnostics but often consider diagnostics a lower priority within the spectrum of STH control efforts. Policymakers were classified as having medium influence and medium interest due to this category’s role in building consensus and reacting to the needs of the STH community. Manufacturers were classified as having medium influence and low interest because they have the capabilities needed to advance STH diagnostics but lack market incentives. Country programs have low influence and medium interest because their desire for improvements in diagnostics depends on other stakeholders to develop products and create a supportive system for implementation. The MDA implementer has low influence and low interest because the role of diagnostics in MDA distribution is poorly defined. After assessing perceived influence and interest, the results distributed stakeholder categories across 4 quadrants, representing varying strategies for communications of the project and the outcomes, none of which have less value and importance. Elements that vary across the groups include frequency, preferred medium, and complexity of communication. “Manage closely” may involve the greatest effort to fully engage and satisfy, “Keep satisfied” may involve enough effort to keep satisfied without boring or overloading, “Keep informed” may involve adequate informing through regular communication to ensure no issues are present with the details of the work, and “Monitor” may involve some but not so much communication as to overload. (Fig 3)

### Key opinion leader interviews

**Fig 4:**
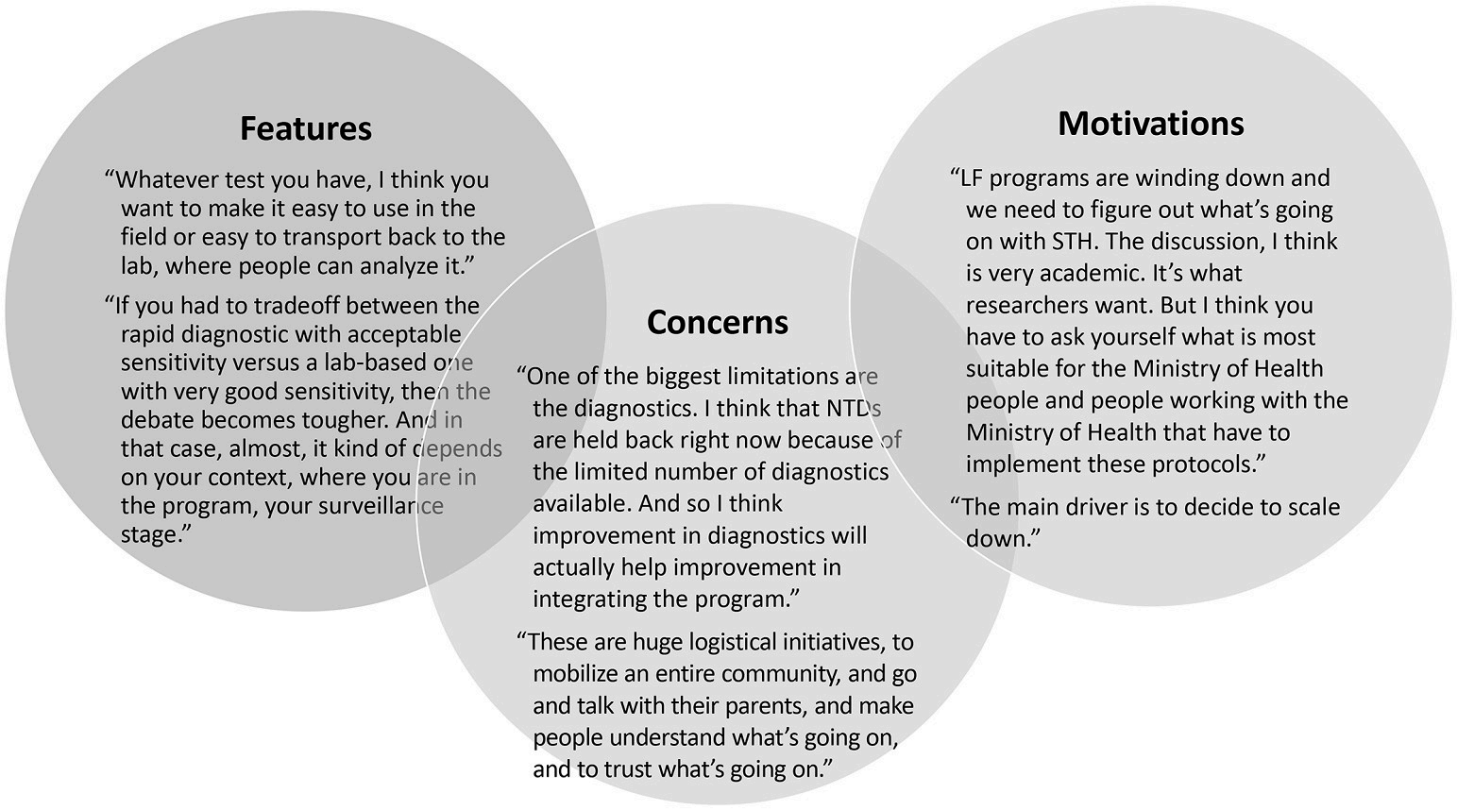
Select findings from key opinion leader interviews.

In total, 10 key opinion leaders were interviewed. Open-ended questions were asked about strengths and weaknesses of current STH surveillance and diagnostics. The reasons for doing STH surveillance were noted as determining baseline measures, especially as LF programs are winding down, as well as for informing decisions to reduce or stop treatment. An interviewee commented that STH infections is a low priority for most donors within the NTD landscape, because STH infections is associated with relatively low morbidity despite high prevalence. Although interviewees acknowledged the limitations of the Kato-Katz method for detecting low-intensity infections, they were generally satisfied with using it for STH surveillance. The attributes of Kato-Katz that were most commented on were that it is well known, easy to use, easy to implement at the local level, and it is a quantitative measurement that can speciate the different STH infections. Some interviewees noted that a test with higher sensitivity than Kato-Katz would be useful. It was also noted that any new test should have a quantitative readout because data on the intensity of infection are used in a variety of ways outside of informing MDA frequency, such as evaluating effectiveness of MDAs, measuring morbidity, and other research purposes. Interviewees commented that a qualitative molecular diagnostic tool may be valuable only when STH prevalence dropped well below 10% to 20%, which represents a small segment of the current market. Several interviewees commented on the feature of easy to use, noting that resource requirements (personnel and infrastructure) necessary for any new test should be like those for Kato-Katz. Highlighted in Fig 4 are select features, motivations and concerns that are useful to consider when guiding the design and implementation of a new STH diagnostic. (Fig 4)

Interviews also explored opportunities and barriers to integration of STH surveillance across the NTDs, and the role diagnostics may play. Achieving sustainable access to new STH diagnostic products through appropriate use and health impact, requires a consideration of the surveillance ecosystem across NTDs because it provides opportunities for synergy, and also challenge in the form of competing interests. Positive attributes were noted by most interviewees such as opportunities for capitalizing on and conserving available resources. However, the challenges associated with integration were also noted. Interviewees noted that implementing change is difficult. Currently, disease control guidelines are separate, agendas are separate, and funding is separate. Although some logistics are similar, such as community-based testing, other activities and required skillsets differ making workforce management more complex. Overall, interviewees considered the integration of STH surveillance with surveillance for other NTDs to be a “nice to have” rather than a high priority.

### Process mapping from observational site visits

Observational site visits in Kenya and the Philippines were conducted by 2 researchers. WHO policies define many procedures of the surveillance activities, such as sentinel site sampling methodologies to select children per school and schools per evaluation unit to ensure prevalence estimates are robust [5,23,26]. Some aspects that vary by location are due to workflow logistics and varying cultural sensitivities by country.

**Fig 5:**
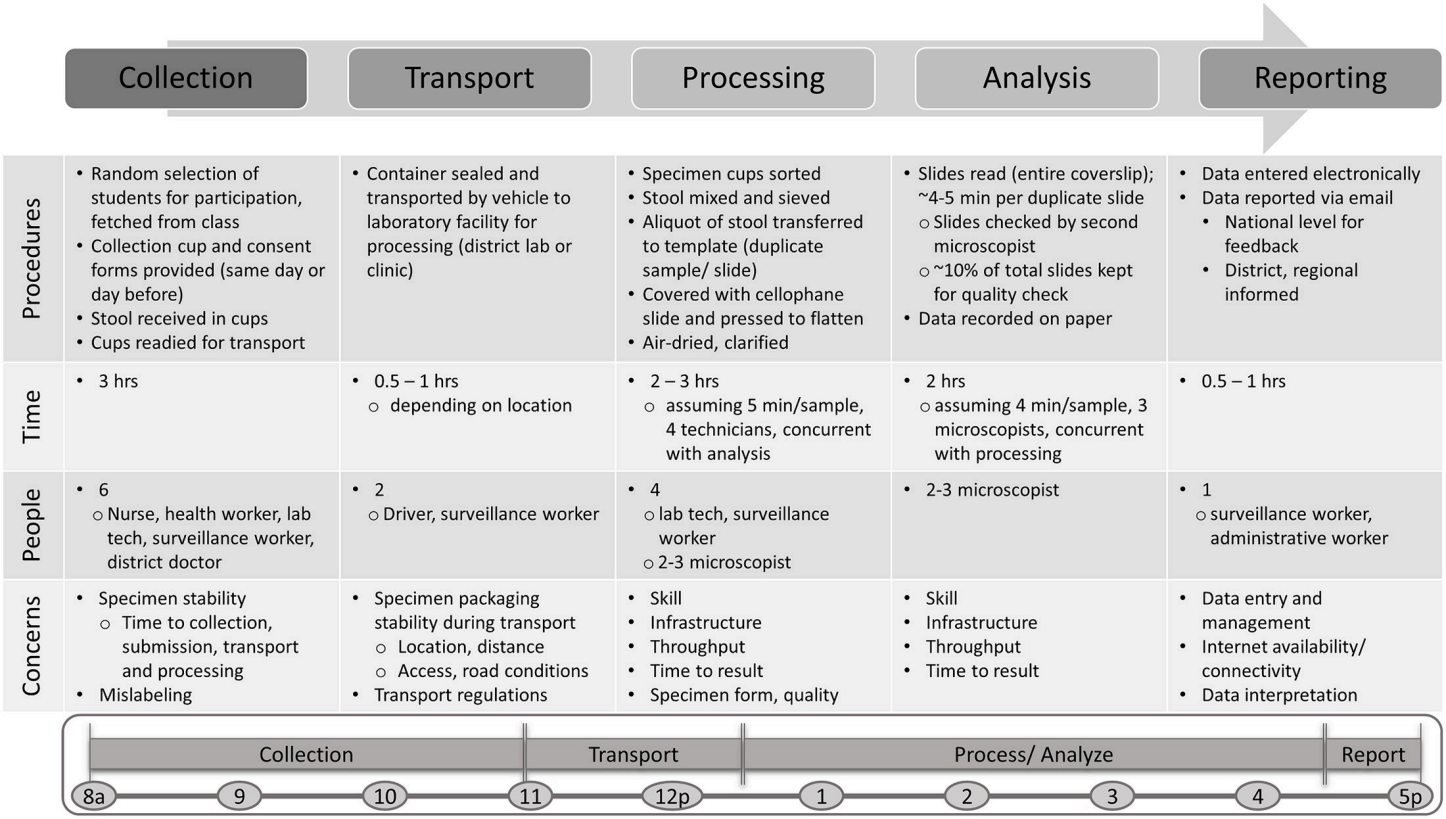
Current workflow of STH surveillance using Kato-Katz microscopy: A summary of similar observations from Kenya and the Philippines.

Each day of surveillance included all necessary procedures for data collection from one school including sample collection, processing, analysis, and reporting. (Fig 5) In the Philippines, collection containers and consent forms were sent home with students the night before allowing parents to help children in sample collection. In Kenya, consent forms were sent home with children prior to sample collection, though collection containers were distributed the morning of the surveillance activity and collected the same day. After filled sample containers were collected from all selected children, specimens were transported to a nearby district lab or clinic facility for processing. Transport time varied day to day as the locations of schools and district facilities were not consistent. All necessary materials were transported by the surveillance teams. The district facilities all had available bench space, electrical supply for a light microscope, and access to water for cleaning slides, though infrastructure and setup varied by location. Processing and analysis were performed concurrently, with several laboratory technicians or surveillance workers preparing the Kato-Katz slides while the microscopists read and recorded the results. The time to read each slide depended on the number and species of eggs detected. After results were recorded on paper forms, data were transferred to electronic forms for dissemination at the national and regional level. At the end of the day, materials were cleaned and repacked for the next day of data collection at another school and district facility. Surveillance teams worked Monday through Friday for several consecutive weeks at a time, before returning to other job functions.

### Quantifying the potential market based on sentinel site monitoring

Data was gathered for 2012 and 2016, to incorporate the newest STH disease burden data available. In 2012, WHO identified that 111 countries required PC for SAC, and in 2016 103 countries required PC for SAC. As a surrogate for STH prevalence, the proportion of SAC requiring PC was calculated by dividing the number of SAC requiring PC by the total number of children between the ages of 5 and 14 years. In 2016, 4 countries with STH burden data did not have disaggregated UN population data (Dominica, Marshall Islands, Nauru, and Tuvalu) so the number of STH tests needed was estimated for 99 countries in 2016. Based on the guidelines, the total SAC population was divided by 200,000 to estimate the number of districts per country [23].

**Fig 6:**
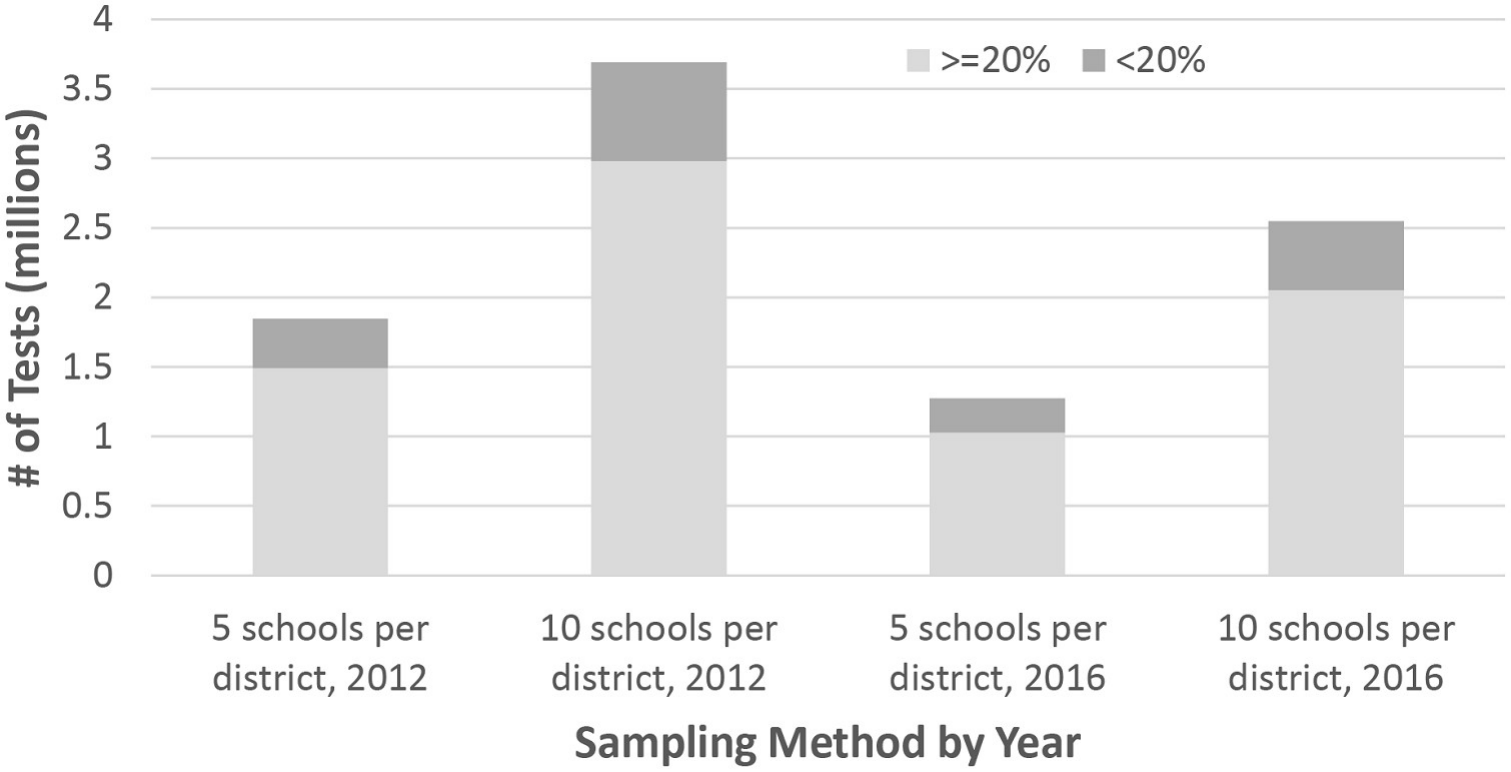
Potential market size for STH surveillance tests estimated for 2012 and 2016. The number of STH tests necessary for surveillance was compared for higher and lower STH burden countries, defined as proportion of SAC population requiring PC above or below 20%.

In 2012, potential market size for all STH surveillance tests ranged from 1.8 to 3.7 million when sampling five or ten schools per district and 50 children per school. Some countries had both high proportions of SAC requiring PC (>25% of school-age children) and high numbers of tests (>100,000 tests). These countries included Bangladesh, Brazil, Ethiopia, India, Indonesia, Nigeria, Pakistan, and the Philippines when sampling 5 schools per district, as well as Colombia, DRC, Kenya, Myanmar, and Tanzania when sampling 10 schools per district. In 2016, potential market size ranged from 1.3 to 2.5 million tests. The remaining high market size countries were India sampled at 5 schools per district, as well as Indonesia, Nigeria, and Pakistan sampled at 10 schools per district. (Fig 6)

To estimate the market size for a diagnostic test that could stop or reduce MDA, the proportion of SAC requiring PC was calculated. WHO guidelines recommend PC for SAC populations with greater than 20% prevalence at baseline. After starting PC, reductions in PC are recommended when the prevalence is less than 20% [23]. The market was estimated for countries with higher STH burden, defined as greater or equal to 20% of SAC requiring PC, or lower STH burden, defined as less than 20% SAC requiring PC. In 2012, there were 29 lower burden countries and 82 higher burden countries, compared to 15 lower burden countries and 84 higher burden countries in 2016. The potential market size in 2012 was 355,000 tests if five schools were sampled and 710,000 tests if ten schools were sampled for lower burden countries, and 1.5 million and 3.7 million tests for higher burden countries. The potential market size in 2016 was 247,000 tests if five schools were sampled and 494,000 tests if ten schools were sampled for lower burden countries, and 1.0 million and 2.1 million tests for higher burden countries.

### Costing analysis

Table 1 shows the results of our analysis comparing the fixed and variable costs of several types of diagnostic tests for STH. These estimates assumed a similar workflow of roughly 100 samples per day. Kato-Katz resulted in the least expensive method at a total cost of $2.29 per patient. FLOTAC was more expensive than Kato-Katz due to consumable costs. The molecular methods were on average 4–6 times the cost of Kato-Katz varying from $7.19 per patient for multiplexed qPCR to $11.91 per patient for multiparallel qPCR. Dx4STH was $9.76 per patient when using a comparable DNA extraction method to the qPCR methods and decreased to $8.33 per patient when a prototype MAL-MB method was used. The largest cost component for molecular methods are consumables, which comprise over 90% of the overall cost. Optimal staffing requirements for all 6 methods were between 3 and 6 staff. For the molecular methods, the time needed to analyse the samples ranged from 4.8 hours for the Simple Dx4STH method and 7.1 hours for the multiparallel qPCR method. When considering the accuracy of the method, the total cost per accurate diagnosis increases for all methods to a cost per patient of $0.06 for Kato-Katz, $0.66 for FLOTAC, $9.01 for Simple Dx4STH, $10.55 for Spin Dx4STH, $12.28 for multiparallel qPCR, and $7.41 for multiplex qPCR. (Table 1)

**Table 1:** Costing analysis for 5 methods to detect STH.

Method	Kato Katz^1^	FLOTAC^1^	Simple Dx4STH	Spin Dx4STH	Multi-parallel qPCR	Multi-plex qPCR
Replicate	2 slides	1 (dual technique)	2	2	2	2
Extraction			MAL-MB^2^	spin column^3^	spin column^3^	spin column^3^
Amplification			isothermal	isothermal	cycler	cycler
Detection Channels			2	2	1	5
# of amplification machines			4	4	2	1
# of staff assuming optimal workflow	4–6	4–6	6	3	6	3
Time to analyze samples (hrs)			4.8	6.0	7.1	5.8
**Cost ($USD) per Patient**						
Instruments	$0.00	$0.00	$0.29	$0.30	$0.69	$0.09
Consumables (extraction)	$0.04	$0.48	$3.68	$5.10	$5.10	$5.10
Consumables (amplification)			$4.37	$4.37	$6.12	$1.99
Sensitivity	50%	50%	90%	90%	95%	95%
Specificity	95%	95%	95%	95%	99%	99%
Accuracy	73%	73%	93%	93%	97%	97%
**Total Cost per Patient**	$0.04	$0.48	$8.33	$9.76	$11.91	$7.19
**Total Consumable Cost per Patient**	$0.04	$0.48	$8.05	$9.46	$11.22	$7.09
**Total Cost per Accurate Diagnosis**	**$0.06**	**$0.66**	**$9.01**	**$10.55**	**$12.28**	**$7.41**

^1^ Source: Speich, *et*. *al*. 2010 [24]

^2^ DNA extraction using a modified alkaline lysis-magnetic bead (MAL-MB).

^3^ DNA extraction using spin column (i.e., QIAamp PowerFecal DNA kit).

#### Willingness-to-pay analysis

**Fig 7:**
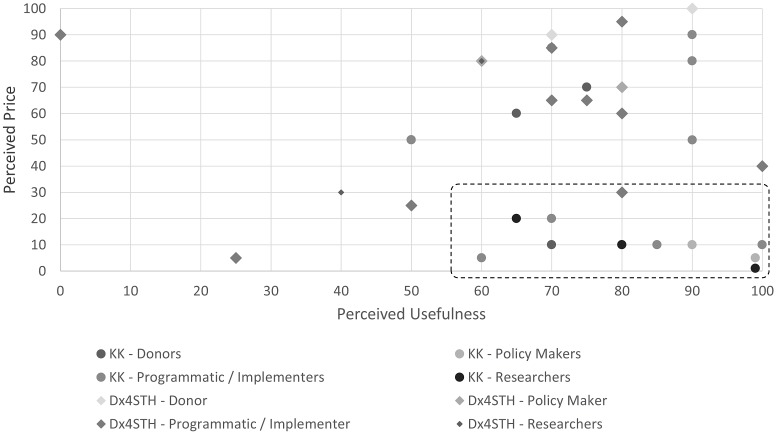
Assessing willingness-to-pay using a perceived value pricing approach: Perceived price compared to perceived usefulness for Kato-Katz and Dx4STH, by stakeholder group.

**Fig 8:**
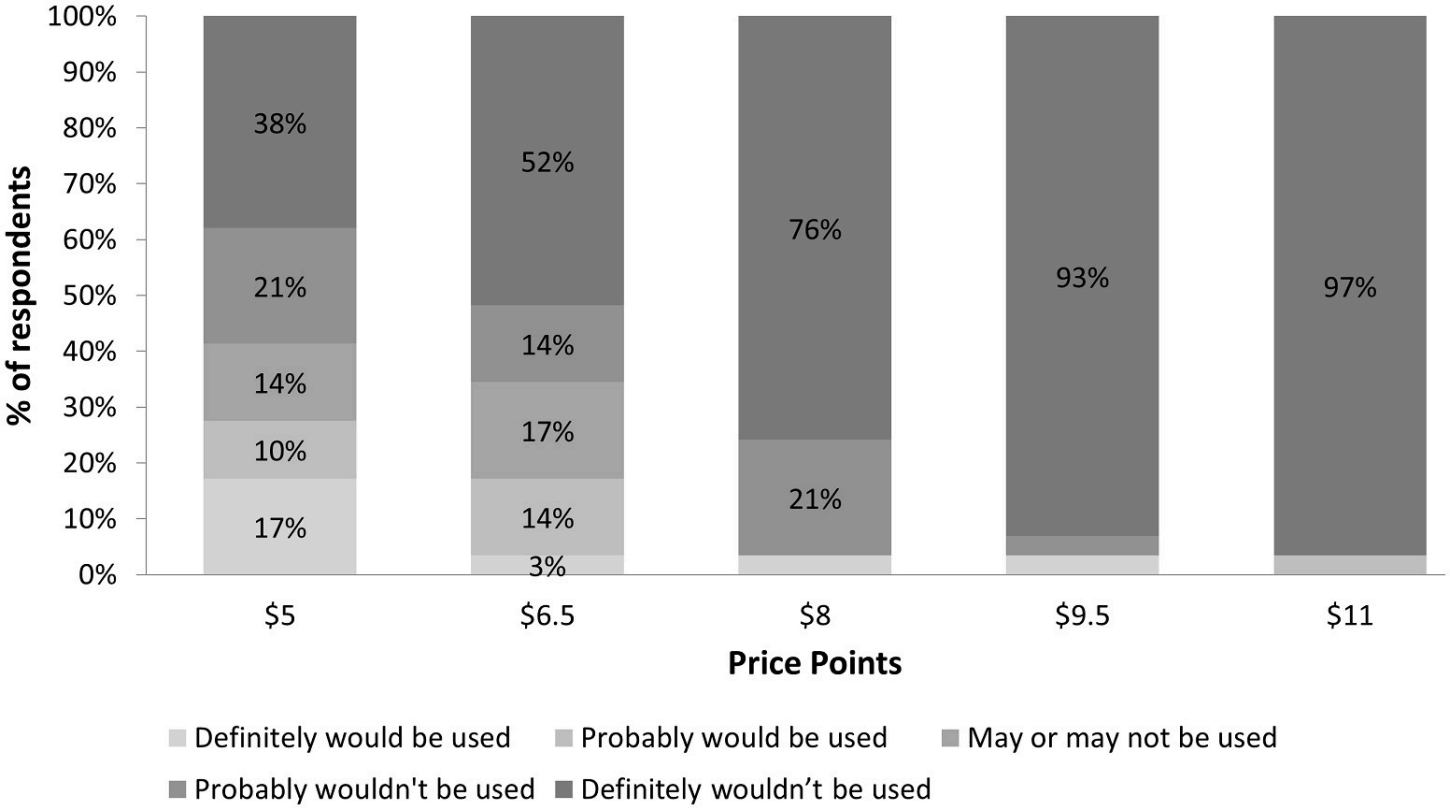
Assessing willingness-to-pay using a Garbo-granger approach: Respondents’ perceptions of the likelihood of surveillance programs to use Dx4STH at each price point. (n = 29).

Using the PVP approach, Kato-Katz had a low perceived price and high perceived usefulness by most stakeholder groups. (Fig 7) Comparatively, Dx4STH had a higher perceived price but comparable perceived usefulness. There was variability in both measures across stakeholder groups. Some respondents indicated the usefulness of both methods would vary by setting, based on prevalence and intensity of infections. Using the Garbo-granger approach, the market for STH diagnostics was determined to be highly price sensitive. Only 27% of respondents said the Dx4STH test would probably or definitely be used at a price of $5 per test, and only 3% thought the test would be used at a price of $11. (Fig 8) Overall, respondents found Kato-Katz to be a useful and cost-effective method despite its well-acknowledged shortcoming, and as a result were unwilling to pay a higher price for it.

## Discussion

In this formative research, an early consideration for user and customer needs, implementation constraints, and market dynamics was used to inform the design and development of a new, more sensitive surveillance test for STH. A focus on access throughout the product lifecycle is central to PATH’s work as a product development partner and is important to achieving health impact through new technologies. Several factors were evaluated here through user and market research, including stakeholder and policy concerns, potential market size, affordability, and acceptability.

Similar to needing broad implementation partnership to achieve the control and elimination goals for STH infections [5], the development and use of new tests for STH will also require broad support across diverse stakeholder groups. Early identification and engagement of key stakeholders is important throughout the product development process. Based on the stakeholder analysis, donors, advocacy programs, and researchers/developers should be engaged with most intensively as they have the most interest and influence in the development of new diagnostics. Obtaining input from manufacturers and policymakers is also key to ensuring sustained access to new diagnostic products in the long term. Specifically, early engagement of manufacturers that can ensure sustained, efficient production and distribution of the final product is particularly critical if meeting reliability and regulatory requirements is a priority. Equally important, country programs should be regularly engaged and informed of progress in diagnostic development as they are the target users, and ultimately, adoption and increased country ownership is dependent on their needs being met [26].

Stakeholder interviews, process mapping, and literature reviews contributed to PATH’s development of target product profiles (TPP) for more sensitive STH surveillance tests in 2015. The process of developing these TPPs for STH, as well as Schistosomiasis and Trachoma, was described in a report (https://www.path.org/resources/diagnostics-ntd/). Since then, global stakeholders meetings on STH diagnostics occurred in 2016 and 2017, namely meetings in Ghent, Belgium and Annecy, France [27]. A challenge considered was balancing the diagnostic needs of the shorter-term goal to monitor progress towards WHO 2020 milestones with the diagnostic needs of the longer-term program goal of transmission interruption. Additional TPP development occurred through a workshop of experts and key opinion leaders, led by the Bill and Melinda Gates Foundation [28]. Of the two TPPs that were developed, the authors noted that one TPP had most of its minimum criteria met by the Kato-Katz method, limiting the role for new diagnostics to addressing the second TPP. Two attributes that Kato-Katz does not meet, however, are stringent regulatory standards (ISO) and quality assurance requirements, which will also be important for any new STH diagnostic and may be addressed through a central laboratory “command center” to ensure unified provision of quality assured diagnostic services [29]. At the STH Advisory Committee meeting in 2017, the qPCR platform was highlighted noting its improved sensitivity in field studies. Standardization, preservation, and DNA extraction methods are the focus of ongoing research. Acknowledgement of the following diagnostic development goals was also stated: increased coordination among partners for improved efficiency, early consideration of field use, focus on test cost and ease of use, and a standardization of techniques [30].

The cost and cost-effectiveness of new diagnostics were important considerations from the beginning of the project. Health impact modeling, performed to understand potential benefits resulting from improvements in diagnostic sensitivity, found that improved diagnostics increase the probability of elimination by a control program through optimized MDA frequency [13]. Interestingly, using more sensitive diagnostics would increase disability adjusted life years (DALYs) averted, and in some scenarios result in an earlier decision to stop MDA, though most often it may result in more frequent MDA. In either case, resources would be more efficiently deployed to improve health impact [13].

The size of the market for high-sensitivity tests may also be a relatively small portion of the overall market for STH diagnostics. In 2016, of the countries requiring PC, 15% had a proportion of SAC requiring PC below 20%. From 2013 to 2016, the number of these lower STH burden countries decreased from 29 to 15, while the higher STH burden countries changed little. Where STH control programs have not yet achieved high MDA coverage, prevalence and intensities of infection may not be reduced to levels low enough to warrant use of more sensitive and expensive molecular tests. The relatively small potential market for high-sensitivity tests may also restrict interest among commercial manufacturers and result in a higher price per test due to the economies of scale. The Dx4STH method would cost somewhere between multiparallel and multiplex qPCR, and all 4 molecular methods were substantially higher than the costs per patient of the copromicroscopic methods. The costing analysis did not include infrastructure or salary costs because comparable estimates were not available for all 6 methods. Most of the cost of Kato-Katz and FLOTAC are salaries [24], so estimates of these methods are artificially low here. However, all 6 methods will have costs due to infrastructure and salaries, though these estimates may vary by method and location. More skilled staff may have higher pay per unit of time. Considering the skill level of the staff, number of personnel for an optimal workflow, and duration of work would impact final costs of a diagnostic method. Additionally, more equipment would add more instrument costs but may also decrease staffing requirements and time. Finally, stakeholders’ willingness to pay for a new molecular test may be lower than the anticipated cost of goods. Interviewed stakeholders believed that donors consider STH infections a lower priority within the NTD landscape due to lower morbidity, potentially influencing perceptions of test value. Cost-effectiveness or cost-benefit analysis will be needed to make the case for use of molecular tools in specific situations.

Overall, the commercial viability of a molecular diagnostic product for STH may be low initially, making donor engagement essential for further product development and introduction. The perceived usefulness of Kato-Katz combined with the higher price for molecular diagnostics contribute to an overall low willingness-to-pay. If initial test volumes needs are low, the absolute cost increase may be acceptable as efficiencies and savings are optimized. The other challenge is that the price sensitivity of the market for new STH diagnostics may be related to the largely donation driven STH control and elimination strategy, which depends on large-scale donation of drugs by pharmaceutical companies, as well as donor dollars for program activities such as implementation, and monitoring and evaluation. The sustainability of long-term dependence on drug donations is uncertain, making the need for innovative, cross-sector drug development partnerships increasingly important [31]. Similarly, innovative partnerships in diagnostic development are also essential to adjust the failed diagnostics market.

During this research, many stakeholders stressed the value of quantitative test results, such as for evaluating treatment effectiveness or estimating morbidity, even though decision-making related to MDA frequency is based on prevalence. Although the Dx4STH test was originally intended to produce only qualitative results to guide decision-making around MDA reductions and stopping [32], stakeholder input led the team to pivot product development to a quantitative test. Compared with a quantitative polymerase chain reaction assay, a quantitative isothermal assay may have the potential to require less expensive and more field friendly equipment while being just as sensitive. Next steps in product development would include further verification testing of the Dx4STH alpha prototype, final development and verification testing of a sample preparation alpha prototype, as well as usability and validation testing in the field. In addition to product development for Dx4STH, further research is also needed to facilitate product adoption and ensure access to any new STH diagnostic, including the development of laboratory protocols for using new tests, additional market research with updated attributes, technology transfer to one or more manufacturers, regulatory and commercialization planning, and implementation research. Broad and inclusive collaboration across the global STH community will make this work more efficient and effective.

There are some limitations to this research. Firstly, the value of formative research is for hypothesis generation. Further evaluative and summative research is necessary to answer questions, some of which may have been highlighted here. Observational site visits were performed in only 2 countries. Differences were identified between these settings, and many more differences are likely to be present when observing more countries. To conduct the market sizing, many assumptions had to be made, such as the frequency of STH surveillance, the distribution of at-risk populations, and the specifics of sentinel site monitoring. The frequency of STH surveillance is unclear as multiple rounds of MDA may be necessary to produce measurable differences. The risk factors for STH infections, such as poor sanitation and hygiene, are not evenly distributed across an entire population of SAC. Therefore, determining the number of districts to include in sentinel site monitoring based on the total school age population may be an overestimate. However, surveillance in other populations such as pre-school age children, women of reproductive age, and community-wide strategies may also adjust the market size. These market size estimates should be updated as new information is available. The costing analysis was based on a combination of published data, process mapping from the observational site visits, and learnings from in house product development for Dx4STH. Assumptions are detailed in supplemental file 2 (S2 File) and could be updated as new information is available. Finally, the willingness-to-pay analysis was conducted when Dx4STH was a qualitative test. Some findings suggest there may be more willingness to pay for a quantitative test, though how much is currently unclear.

The need for new, more sensitive STH diagnostics has been recognized for some time, and many groups have made substantial progress in developing these new technologies. Formative research has supported PATH’s product development of a new molecular diagnostic kit that addresses user needs and implementation requirements while laying the groundwork for future access. Some barriers to moving further include the need for funding to support continued development and evaluation, as well as revisions to policies for STH surveillance to optimally support the use of newer technologies [26]. The inertia created by the cost-effectiveness of MDA with currently donated drugs and the functioning of the Kato-Katz method up to this point, make changing the status quo more challenging. The desire may be to create high sensitivity tests that function within existing guidelines and still maintain the low cost, low complexity, minimal infrastructure requirements expected from decades of using Kato-Katz. Reconsidering this expectation may speed progress to improve program decision-making, and ultimately, eliminate disease.

## Supporting information

S1 File Market size analysis. (XLSX)

S2 File Costing analysis. (XLSX)
